# First case report of cerebral folate deficiency caused by a novel mutation of *FOLR1* gene in a Chinese patient

**DOI:** 10.1186/s12881-020-01162-3

**Published:** 2020-11-26

**Authors:** Ciliu Zhang, Xiaolu Deng, Yafei Wen, Fang He, Fei Yin, Jing Peng

**Affiliations:** 1 Xiangya Hospital Central South University, 87 Xiangya Road, Changsha, Hunan 410008 P.R. China; 2 XiangYa School of Medicine of Central South University, 172 Tongzipo Road, Changsha, Hunan 410013 P.R. China

**Keywords:** Seizures, *FOLR1*, 5-MTHF, Calcium folinate

## Abstract

**Background:**

Cerebral folate deficiency (CFD) is a neurological disease, hallmarked by remarkable low concentrations of 5-methyltetrahydrofolic acid (5-MTHF) in cerebrospinal fluid (CSF). The primary causes of CFD include the presence of folate receptor (FR) autoantibodies, defects of FR encoding gene *FOLR1*, mitochondrial diseases and congenital abnormalities in folate metabolism.

**Case presentation:**

Here we first present a Chinese male CFD patient whose seizure onset at 2 years old with convulsive status epilepticus. Magnetic Resonance Imaging (MRI) revealed the development of encephalomalacia, laminar necrosis in multiple lobes of the brain and cerebellar atrophy. Whole Exome Sequencing (WES) uncovered a homozygous missense variant of c.524G > T (p.C175F) in *FOLR1* gene. Further laboratory tests demonstrated the extremely low level of 5-MTHF in the CSF from this patient, which was attributed to cerebral folate transport deficiency. Following the intravenous and oral treatment of calcium folinate, the concentrations of 5-MTHF in CSF were recovered to the normal range and seizure symptoms were relieved as well.

**Conclusions:**

One novel variation of *FOLR1* was firstly identified from a Chinese male patient with tonic-clonic seizures, developmental delay, and ataxia. The WES and laboratory results elucidated the etiology of the symptoms. Clinical outcomes were improved by early diagnosis and proper treatment.

## Background

As defined in 2004 by Ramaekers and Blau, Cerebral Folate Deficiency (CFD) encompasses a collection of neurological syndromes associated with low cerebrospinal fluid (CSF) concentrations of 5-methyltetrahydrofolate (5-MTHF), the normal folate metabolism in nervous system [1]. The circumstantial evidence show that CFD is caused by congenital metabolic disorders and the acquired folic acid deficiency [2, 3]. The most common cause of CFD is the presence of folate receptor (FR) autoantibodies [4]. In addition, defects of FR encoding gene *FOLR1*, mitochondrial diseases and congenital abnormalities in folate metabolism could also lead to CFD. *FOLR1* (OMIM#613068), located in the long arm of chromosome 11, encodes for folate receptor α (FRα) [5]. Binding of FRα with 5-MTHF in high affinity is essential for the transportation of folate to the brain [6, 7]. Thus, pathogenic variants of *FOLR1* could result in brain-specific 5-MTHF deficiency, and ultimately cause a series of neuropsychiatric symptoms [5]. Normally, the onset of CFD caused by mutated *FOLR1* begins from late infancy [8], with the major clinical manifestations including developmental retardation, dyskinesia, epileptic seizure, leukodystrophy and slow EEG background activity. In infant patients, 5-MTHF concentration in cerebrospinal fluid is also extremely low, but remains normal in peripheral nervous system, indicating that the cerebral 5-MTHF deficiency is the underlying mechanism for infant CFD.

## Case presentation

### Patient

The Chinese patient was born at full term through normal vaginal delivery, weighting 3.25 kg that fell in the normal range of newborn weights. He showed developmental delay and mental retardation since one and half years old. He was able to raise his head at 3 months, sit at 6 ~ 7 months, walk at 24 months, and learn limited vocabulary at 12 months. The patient was in poor physical health since he was 2 years old.

At 2 years of age, the patient started to have tonic-clonic seizures that lasted for 20–30 min with high fever (39 degrees Celsius) and was initially diagnosed as febrile seizure at local hospital. The patient was treated with anticonvulsive medications. The symptoms were relieved afterwards. However, a similar yet more severe symptoms appeared around age of four. Following similar treatments, the patient was transferred to another hospital to seek out a detailed clinical examination. CT scan of brain did not detect lesions. The patient was subsequently diagnosed with viral meningitis treated with anti-virus medicine. At the age of 6 years and 9 months, the symptom relapsed. The patient manifested vomiting, headache, tonic-clonic seizure, symptomatic generalized epilepsy that could last for half an hour. The patient was intubated and treated with the combination of antibiotics, phenobarbitone and sodium valproate.

Although the symptoms were relieved to some extent when the patient took sodium valproate at dose of 0.2 g Tid (26 mg/kg/d), the etiology of the symptoms remained unknown. Therefore, at age of 6 years and 11 months, after about 4 years of repeatedly suffering from status epilepticus, the patient was referred to Xiangya Hospital, Central South University, Hunan Province, China. The detailed neurological exams showed that the patient clinically manifested global developmental delay, slurred speech, hyperactivity, sialorrhea, difficulty in managing behavior, unsteady gait, hypotonia. His head circumference was 46 cm (less than the 3rd percentile). An extensive laboratory and genetic analysis were performed to uncover the etiology of his disease.

**Fig. 1:**
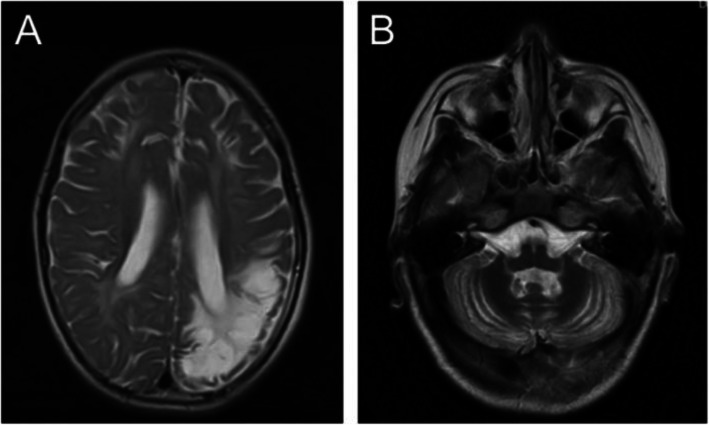
Enhanced Brain MRI of a 6 years and 11 months old male patient. **a** MRI image of brain scan showed encephalomalacia and laminar necrosis with diffuse white matter disorder. **b** MRI scan of the brain demonstrated cerebellar atrophy in the patient

We performed brain MRI, diffusion weighted imaging (DWI). First, we found encephalomalacia and laminar necrosis in the brain left parietotemporal lobe, hippocampus and bilateral frontal lobe with diffuse white matter disorder (Fig. 1a). Second, cerebellar atrophy was also diagnosed from the brain scan (Fig. 1b). No abnormality was identified in the craniocerebral MRA scan. Clinical data of the family were collected and analyzed under the approval by the Ethics Committee Xiangya Hospital, Central South University. Informed consent was obtained from the parents of this proband.

### Genetic analysis

Whole exome sequencing (WES) was performed to search for potential pathogenic variants in an unbiased way. Genomic DNA was extracted from peripheral blood of the proband and his parents as previously described [8]. The putative mutations in Genomic DNA were subsequently screened by WES. Sequence variants were checked with population databases gnomAD (http://gnomad.broadinstitute.org/) and evaluated using multiple bioinformatic programs. Variant pathogenicity was interpreted according to the American College of Medical Genetics (ACMG) guidelines [9]. The variant was further confirmed by Sanger sequencing.

**Fig. 2:**
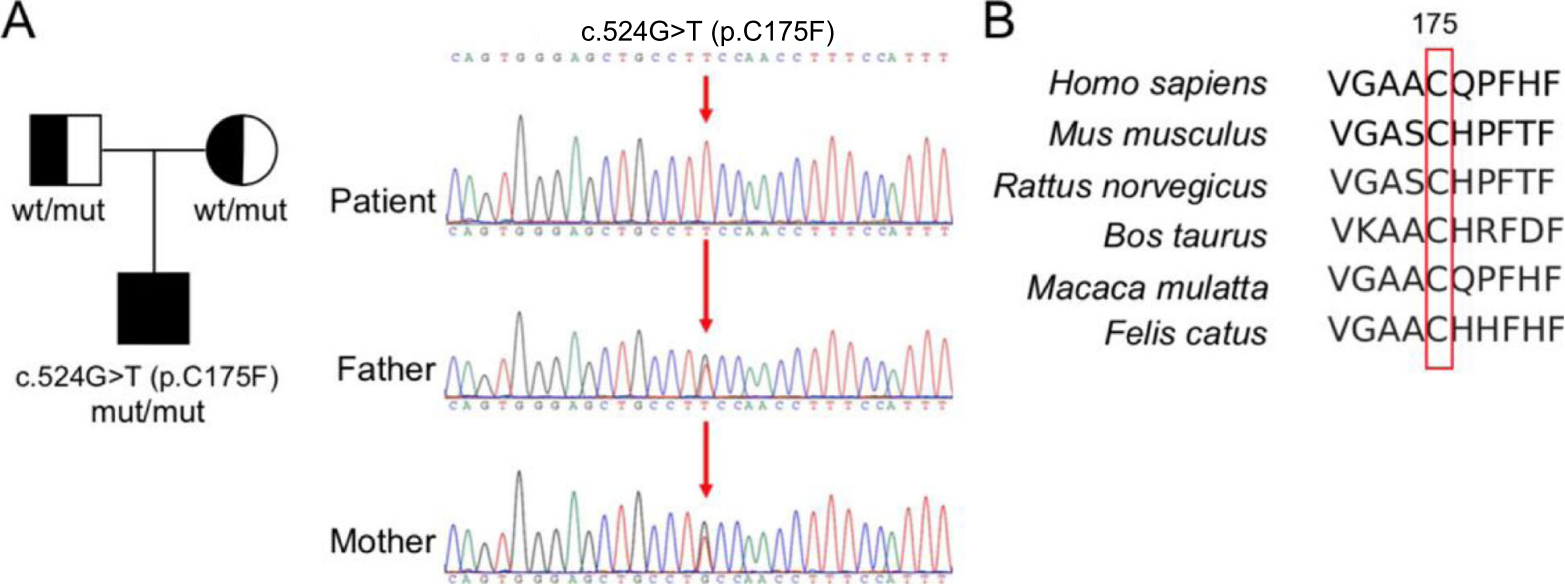
Whole-exome sequencing (WES) and Sanger sequencing revealed missense variant of c.524G > T (p.C175F) in *FOLR1* gene. **a** Homozygous variant in proband and heterozygous variant in his parents were identified. **b** Evolutionary conservation of cysteine residue at position 175 (red box) in the *FOLR1* gene among species. wt, wildtype

A novel variant c.524G > T (p.C175F) in folate receptor alpha (*FOLR1*) was identified. The Sanger sequencing confirmed the homozygous state of the variant in the affected individual, with the parents of being the heterozygous carrier (Fig. 2a). The variant was absent from population database gnomAD. Multiple bioinformatic analysis results (Polyphen2 score 0.995, probably damaging; Mutation Taster score 0.998, disease causing; and SIFT score 0, deleterious) indicated that the variant was deleterious.

### Further diagnostic assessment

Laboratory blood routine tests revealed the total folate was 2.20 μg/L, lower than the minimal normal density of (3 μg/L) and the intracellular folate was 82.66 μg/L, lower than minimal normal range (93 μg/L).

Further examination revealed that the patient had almost undetectable levels of CSF 5-MTHF. The average concentration of 5-MTHF was 1.38 nmol/L, which is dramatically lower than the normal concentrations (60 ~ 210 nmol/L) for 6 ~ 15 years old children. The results indicated the cerebral folate deficiency. Taken together, laboratory results and WES analysis demonstrated that the mutation in *FOLR1* and therefore the CSF 5-MTHF deficiency serves as the major etiology of patient’s symptoms.

### Treatment

We devised the following treatment procedures based on genetic and laboratory test results: calcium folinate was given to the patient by intravenous (iv) injection at dosage of 2 mg/kg/d for 1 week, and adjusted to 6 mg/kg/d by oral administration, and gradually increased to 11 mg/kg/d orally at last. The CSF-5MTHF concentration was monitored closely during the treatment with calcium folinate. We gradually reduced the supplement of antiepileptic drugs (AEDs) until the patient was seizure free at the end. After 2 months of treatment, the CSF 5-MTHF concentration was elevated to 36.24 nmol/L, and 6 months later, the CSF 5-MTHF concentration was increased to 78.76 nmol/L that fell into the normal range of 60 ~ 210 nmol/L for 6 ~ 15 years old children. Besides, the symptoms including unsteady gait, failure to hold objects readily, epileptic seizure, etc. were considerably improved. Furthermore, after the treatment, seizure no longer concomitantly happened with fever.

## Conclusions and discussion

In this report, we documented a first case of CFD patient in China caused by a novel mutation of *FOLR1* gene (Fig. 1a). The young patient had been transferred between hospitals and the symptoms relapsed frequently prior to the elucidation of the underlying cause of his symptoms. The elaboration of the association between the mutation of *FOLR1* gene and the clinical manifestations greatly expedited the treatment of CFD in this case.

FOLR1 is glycophosphatidylinositol (GPI)-anchored cell membrane protein, which regulates folate transport into the cells [6]. Chen et al. studied the crystal structure of human FOLR1 in complex with folic acid at 2.8 Å resolution [7]. Another group determined discrete structural conformations dependent on pH changes [10]. No direct evidence has been found to support that the novel variant c.524G > T (p.C175F) is involved in ligand binding. However, C175 is conserved among different species, and clinical data shown in this study demonstrated the importance of this residue. Possibly, mutation of this site will somehow sabotage the protein function. The further experiments will shed light on how this variant affects FOLR1 function as well as folate binding ability.

**Fig. 3:**
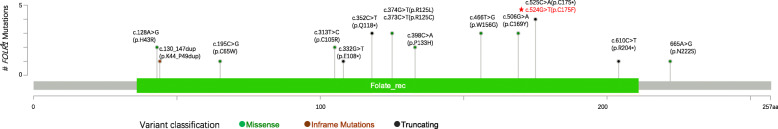
Lollipop graph shows mutations in FOLR1 gene reported in literatures. Red star indicates the variant identified in this study. Green dots: missense mutation; Red dots: inframe mutation; Blue dots: Truncating mutation. Green bar: Folate receptor family domain where folate binds. Note: g.3576 T > G splice mutation (from ref. [8]) is not included in this graph due to its being out of the scope after re-annotation

Pathogenic variants attributed to neurological disorders have been recovered in *FOLR1* gene locus [2, 5, 8, 10, 11]. Homozygous mutations or compound heterozygous mutations led to autosomal recessive disorders, while a homozygous 18-bp in-frame duplication in *FOLR1* gene also linked to neurodegeneration [5]. It transpires that patients carrying *FOLR1* mutations exclusively end up with substantial low concentrations of CSF 5-MTHF (≤ 5 nmol/L), which lead to multiple brain lesions as revealed by neuroimaging studies. By reviewing pertinent literatures, we summarized all existing 15 variants from 23 patients in Fig. 3 [2, 5, 8, 10, 12–17]. In this case, we added a novel homozygous p.C175F variant in *FOLR1* genotype spectrum. Alignment of the protein sequences of human *FOLR1* with those from other organisms shows that the residue C175 is conserved across different species (Fig. 2b), implying that this site is critical for normal protein function and mutant protein may be defective in 5-MTHF binding and translocation. To improve the low concentrations of 5-MTHF caused by p.C175F variant, we deployed calcium folinate, the racemic stable form of folate, through oral or iv administration [18]. The treatment regime alleviated the symptoms efficiently. Given that the overdose of folic acid treatment could cause neurotoxicity [19], it is imperative to distinguish folinic acid from folic acid, and the latter may deteriorate the patient symptoms by sequestration of folic receptors through tight binding. In this respect, we meticulously monitored the changes in CSF 5-MTHF concentrations to avoid any side effect of the treatment.

In conclusion, the understanding of the genetic basis of frequently relapsed epileptic syndrome is critical for devising an effective treatment. This could be a tremendous benefit to the young patient, because the earlier the effective treatment is applied, the better recovery can be.

## Data Availability

The data that support the findings of this study are available from the corresponding author upon reasonable request. Data has been deposited and is publicly available in NCBI SRA database at weblink: https://www.ncbi.nlm.nih.gov/Traces/study/?acc=PRJNA672777

## Abbreviations

CFD: Cerebral folate deficiency
5-MTHF: 5-methyltetrahydrofolic acid
CSF: Cerebrospinal fluid
FR: Folate receptor
MRI: Magnetic Resonance Imaging
WES: Whole Exome Sequencing
DWI: Diffusion weighted imaging
ACMG: American College of Medical Genetics
AEDs: Antiepileptic drugs
